# Rewiring the *Drosophila* Brain With Genetic Manipulations in Neural Lineages

**DOI:** 10.3389/fnmol.2019.00082

**Published:** 2019-04-04

**Authors:** Luis F. Sullivan

**Affiliations:** Institute of Neuroscience, University of Oregon, Eugene, OR, United States

**Keywords:** *Drosophila*, neuroblasts, spatial patterning, temporal patterning, Notch-signaling, neural diversity, circuits, central complex

## Abstract

Neurons originate from neural stem cells and then synapse with stereotyped partners to form neuronal circuits. Recent findings indicate that several molecular mechanisms generating neuronal identity can rewire neuronal connectivity in the *Drosophila* brain when genetically manipulated. In this review, I discuss how mechanisms generating neuronal identity could activate molecular pathways essential for circuit formation and function. Next, I propose that the central complex of *Drosophila*, an ancient and highly conserved brain region essential for locomotor control and navigation, is an excellent model system to further explore mechanisms linking circuit development to circuit function.

## Brief Historical Perspective

Neurons have diverse functions in the brain, ranging from post-sensory processing to subsequent computations for behavior (Shadlen et al., 1996; Gold and Shadlen, 2001). To function properly, neurons must assemble into complex, anatomically stereotyped circuits. Such anatomical circuits or maps require precise dendritic patterning, axonal targeting, and synapse partnering. Each component of **circuit development** (see Key Concepts) requires a range of molecular and genetic mechanisms, from cell-surface molecules to transcription factors (Dickson, 2003; Tada and Sheng, 2006; Williams et al., 2010; Enriquez et al., 2015). Neurons can express these genes differently over time, which likely accounts for much of their anatomical and functional diversity in the adult brain. One possibility is that the mechanisms that establish molecular diversity in the brain during neurogenesis are independent of those that establish connectivity. However, from work in *Drosophila*, a picture emerges where molecular mechanisms during neurogenesis can rewire precise circuit anatomy when genetically manipulated, which implicates genes generating **neuronal identity** as direct regulators of circuit assembly (Kao et al., 2012; Sen et al., 2014; Pinto-Teixeira et al., 2018).

*Drosophila* neural progenitors are exposed to specific factors across both spatial and temporal axes. Initially, as ventral nerve cord (VNC) progenitors delaminate from the neuroepithelium they are exposed to *spatial* *cues* based on where they delaminate (Truman and Bate, 1988; Skeath and Thor, 2003). From here, they are exposed to *temporal cues* as they age and generate neural progeny (Doe, 2017). Finally, as ganglion-mother cells (GMCs; the direct progeny of neuroblasts) divide symmetrically into two distinct neural progeny, they generate neurons that are either *Notch-On* (N^ON^) or *Notch-Off* (N^OFF^), forming two distinct “hemilineages” from a single neural progenitor (Truman et al., 2010). Each of these mechanisms during neurogenesis is essential for generating **neural diversity** in the adult brain. Recently, these mechanisms have been correlated with the assembly of neuronal circuits, implicating a link between neural diversity and neural circuits (Kao et al., 2012; Sen et al., 2014; Pinto-Teixeira et al., 2018). For this review, I summarize the genetic manipulations that can rewire the *Drosophila* brain, and propose that the central complex of *Drosophila* is an excellent model system to determine basic developmental mechanisms essential for circuit function and animal behavior.

KEY CONCEPT 1 **Circuit development**

During animal development, neurons connect with other neurons to signal electrical or chemical information across the synapse. Together, as neurons develop many more connections with multiple partners, circuits emerge to process and integrate information across many sensory or motor modalities for animal behavior.

KEY CONCEPT 2 **Neuronal identity**

Each neuron has a relatively unique molecular, anatomical, and physiological features in the brain. This profile is how we identify single types or groups of neurons that share common features in the brain.

KEY CONCEPT 3 **Neural diversity**

While each neuron or group of neurons has a unique identity, the sum of these identities comprises the brain. A brain’s degree of neural diversity refers to the total number of neuronal cell types that comprise it. Simpler brains yield fewer cell types than more complex brains, such as the neocortex.

## Summary of the Established Principles

### Spatial Genes and the Assembly of Neural Circuits

**Figure 1:**
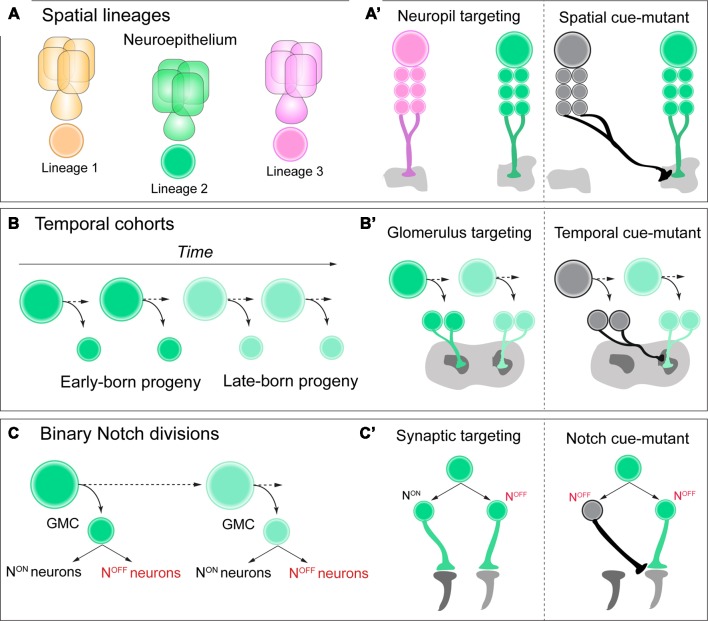
**(A,A’)** Neuroblasts acquire a spatial identity based on where they delaminate from the neuroepithelium. Each spatial identity then generates a unique lineage. Each lineage has unique neuropil targeting in the adult central brain of *Drosophila*, and when these spatial identity genes are mutated in each lineage, this neuropil targeting can be transformed. **(B,B’)** As neuroblasts generate neural progeny and age, they express a series of temporal identity genes, establishing cohorts of neural progeny over time, such as early vs. late-born neurons. These neurons have distinct glomerulus targeting in the adult antennal lobe, and if specific temporal identity genes are mutated, this targeting can be transformed. **(C,C’)** As ganglion-mother cells (GMCs) divide, they generate two distinct Notch ON vs. OFF “hemilineages” in the adult brain. These hemilineages can have very distinct synaptic targeting in the optic lobe of *Drosophila*, and if Notch signaling is mutated, their connectivity can be transformed. See text for details and references.

Tens of thousands of neurons within the *Drosophila* central brain emerge from a relatively small pool of ~100 neural progenitors (Truman and Bate, 1988; Urbach, 2003; Technau et al., 2006). Neurons from the same **neuroblast lineage** often share anatomical and functional features of connectivity by innervating common neuropil regions or axon tracts within the central nervous system (Pereanu and Hartenstein, 2006; Ito et al., 2013; Lovick et al., 2013; Yu et al., 2013; Figure 1A). During vertebrate cortical development, neurons that are clonally related commonly innervate the same column or exhibit similar functional properties in response to external stimuli (Yu et al., 2009; Li et al., 2012; Ohtsuki et al., 2012). Altogether, for both invertebrate and vertebrate species, lineages are a core determinant of neuronal circuit assembly.

During embryogenesis *Drosophila* neural stem cells, called neuroblasts, are exposed to spatial genes as they delaminate from the neuroepithelium. Each spatial gene across both the anterior-posterior and dorsal-ventral body axes conveys unique molecular information for each progenitor, establishing the molecular identity of neural progeny generated by each lineage (Skeath and Thor, 2003; Urbach and Technau, 2004; Technau et al., 2006), a common feature for both vertebrate and invertebrate neural patterning (Reichert and Simeone, 2001; Lichtneckert and Reichert, 2005; Reichert, 2009). Until recently, it was unclear whether these unique genetic programs, which confer progenitor heterogeneity, are also involved in the assembly of complex circuit anatomy and function.

KEY CONCEPT 4 **Neuroblast lineage**

The initial neural stem cell generating neurons or glia in a particular region of the brain in insects is referred to as a neuroblast. Neural progeny that originate from the same parental stem cell are clonally related, or daughter cells of the same lineage.

Within the diverse network of adult brain lineages in *Drosophila*, the LALv1 and ALad1 neuroblast lineages are both molecularly and anatomically distinct. LALv1 projects to the central complex, a dense neuropil region associated with adult navigation (Pfeiffer and Homberg, 2014), whereas ALad1 projects to the antennal lobe, where olfactory information is processed (Fishilevich and Vosshall, 2005). Sen et al. (2014). demonstrate that a single spatial factor, *orthdenticle* (otd), is expressed in LALv1 but not ALad1. When this factor is mutated with clonal analysis, LALv1 lineage tracts adopt the same projection pattern as ALad1, and project to the antennal lobe. Conversely, misexpression of otd in ALad1 causes a partial reciprocal transformation of connectivity to the central complex. Finally, otd-mutant LALv1 neurons are functionally integrated into antennal lobe circuitry, and process olfactory information much like ALad1 lineage neurons (Sen et al., 2014). Altogether, these data demonstrate that spatial identity during neurogenesis in the *Drosophila* brain can transform and regulate functional neuronal connectivity, or macro-neuroanatomy (Figure 1A’).

### Temporal Genes and the Assembly of Neural Circuits

Neurons from a common lineage share many features of connectivity, such as innervating a common neuropil structure. Yet within each neuropil boundary, there are substructures or targeted regions of innervation, such as glomeruli or layers (Couto et al., 2005; Wolff et al., 2015). It has been shown in the *Drosophila* antennal lobe that neurons innervate each glomerulus according to their birth-order from a neuroblast lineage (Jefferis et al., 2001). This pioneering study demonstrated that mechanisms regulating neuronal birth-order can determine which glomerulus a neuron will innervate. It could be that neurons encounter different extrinsic cues and environments based on their birth-order, or that intrinsic factors such as temporal identity genes instruct circuit assembly. For vertebrates, mammalian cortical neurons innervate distinct layers of the cerebral cortex based on their birth-order from radial glia progenitors (Molyneaux et al., 2007; Leone et al., 2008). Together, from vertebrates to invertebrates, precisely timed neurogenesis is potentially a powerful mechanism for determining which substructure neural progeny will innervate from a lineage. It is unclear, however, whether this detailed circuit assembly is directly linked to birth-time, temporal identity, or both.

Temporal genes have been shown to regulate neurogenesis based on birth-order across both mammalian and insect species (Kohwi and Doe, 2013; Figure 1B). For *Drosophila*, neuroblasts that generate projection neurons of the antennal lobe express the transcription factor *chinmo* early during larval life (Zhu et al., 2006). When this early temporal transcription factor is mutated from antennal lobe lineages with clonal analysis, neurons that are early-born now target late-born glomeruli in the antennal lobe, essentially transforming their glomerulus targeting (Kao et al., 2012; Figure 1B’). Future work in both *Drosophila* and vertebrate species could determine if this is a universal mechanism across various stages of brain development, rather than unique to the antennal lobe of the adult fly.

### Notch Signaling and the Assembly of Neural Circuits

To assemble functional circuitry coordinating behavior, single neuronal cell types must synapse with specific partners with stringent specificity, and this conferred by either local guidance cues or synaptic specification molecules (Benson et al., 2001; Betley et al., 2009; Williams et al., 2010). This detailed level of circuit formation is still poorly understood, and the mechanisms are still under heavy investigation. It remains clear that single neuronal cell types position their axons and dendrites in highly distinct regions of neuropil structures, this is called synaptic targeting. Without highly arrayed structures, such as retinotopic maps in the optic lobe of *Drosophila* or mammalian visual cortex, animals would be unable to robustly and routinely process complex stimuli, such as primitive motion detection of objects in the visual-spatial world (Melnattur and Lee, 2011; Zhuang et al., 2017).

A recent study discovered that the retinotopic map of adult *Drosophila* required temporal patterning and Notch signaling to correctly organize specific lobula cell-type neurons within a circuit map for motion detection (Pinto-Teixeira et al., 2018). T4/T5 motion detection neurons are generated by the same GMC, with Notch^OFF^/Notch^ON^ generating each subtype, respectively. When Notch-cues are mutated from this GMC with clonal analysis, both neurons are now T4 identity with identical morphology and targeting (Figures 1C,C’). This study highlights the power of *Drosophila* genetics to uncover simple and basic rules during development that can govern the organization of complex circuit topography.

### Current State of the Art

Molecular cues used to guide neurogenesis (spatial, temporal, and Notch signaling) correlate with the assembly of neuronal circuits, yet few studies have directly demonstrated that these cues activate genes directly regulating synaptic connectivity, such as cell-surface molecules. One pioneering study discovered that axon trajectory choice in the antennal lobe of *Drosophila* was controlled by both Notch signaling and the subsequent expression of semaphorin protein, a cell-surface molecule known for its role in axon guidance. Notch mutants were characterized as having the same axon trajectory choice defects as semaphorin mutants for antennal lobe projections (Joo et al., 2013). In order to guide subsequent post-mitotic neurons to their correct neuropil, glomerulus, or synaptic target, spatial or temporal patterning cues could activate similar molecular mechanisms. With the advent of single-cell transcriptomics, these molecular mechanisms could be readily identified and tested in simple nervous systems such as *Drosophila*, as well as other model organisms.

## Highlight of Future Directions

Many studies highlight the importance of specific genes, such as cell-surface molecules and cytoskeletal regulators, for circuit development, but few of these have been directly linked to circuit function and ultimately to animal behavior (Sullivan et al., 2019). The primary challenge is that behaviors, such as locomotion or vision, are often robust, with redundant or parallel pathways that compensate for minor defects or mutations to single neuronal cell-types. A potential way to overcome these challenges is to investigate behaviors that rely on neural circuit “bottlenecks”—regions where information flow critical to a particular behavior converges onto a small group of neurons (Olsen and Wilson, 2008).

One brain region in invertebrates that will likely prove sensitive to many developmental defects is a highly conserved brain region in arthropods, termed the central complex, positioned along the midline of the adult brain. The behavioral correlates of this region include path-integration, celestial navigation, sleep, and general sensorimotor transformations (Seelig and Jayaraman, 2015; Pimentel et al., 2016; Giraldo et al., 2018). These behaviors are critical for animal survival; they rely on very specific subsets of neurons within the central complex, many of which form “bottlenecks” where information must flow through a single class of neurons (Franconville et al., 2018). These “bottlenecks” could be overcome by finding the genetic mutations required to assemble these neurons into circuits. These will likely yield robust behavioral deficits that are readily quantified and are independent of basic sensory or motor systems such as vision and locomotion. Taken together, while much of the behavioral neuroscience community is focused on central complex function, the community of developmental neuroscientists could begin to investigate how this highly conserved brain region assembles its precise circuit anatomy. We can bridge a gap that is often dividing our field into two areas without much overlap and ultimately discover how a circuit develops to drive animal behavior.

Mechanisms that expand neural diversity during neurogenesis have been well characterized for the last two decades in *Drosophila* and vertebrate species. Post-mitotic mechanisms that regulate neuronal connectivity have also been well characterized. To date, there are few examples linking these two areas of developmental neuroscience together. Are the mechanisms that regulate molecular diversity also required to regulate neuronal connectivity? It could be that these two areas are independent and that cell-surface proteins operate only after mitosis. Alternatively, the initial genes activating cell-surface molecule expression could begin with spatial identity, temporal identity, or Notch-signaling during neurogenesis. Future work in relatively simple model organisms, such as *Drosophila*, could yield valuable insights into this emerging area in developmental neuroscience.

## Author Contributions

LS conceived and wrote this manuscript, and constructed the figure.

## Conflict of Interest Statement

The author declares that the research was conducted in the absence of any commercial or financial relationships that could be construed as a potential conflict of interest.
